# Acupuncture for Primary Dysmenorrhea: An Overview of Systematic Reviews

**DOI:** 10.1155/2018/8791538

**Published:** 2018-11-21

**Authors:** Furong Zhang, Mingsheng Sun, Shanshan Han, Xiaoyu Shen, Yanan Luo, Dongling Zhong, Xiujuan Zhou, Fanrong Liang, Rongjiang Jin

**Affiliations:** ^1^College of Health Preservation and Rehabilitation, Chengdu University of Traditional Chinese Medicine, Chengdu 610075, China; ^2^College of Acupuncture and Tuina, Chengdu University of Traditional Chinese Medicine, Chengdu 610075, China; ^3^College of Clinical Medicine, Chengdu University of Traditional Chinese Medicine, Chengdu 610075, China; ^4^Nuclear Industry 416 Hospital, Chengdu 610075, China

## Abstract

**Objectives:**

As current evidence of the effectiveness on acupuncture for primary dysmenorrhea (PD) is inconsistent, we aimed to critically appraise the evidence from relevant systematic reviews (SRs).

**Methods:**

SRs of randomized controlled trials (RCTs) concerning acupuncture and PD were searched in four databases. The Preferred Reporting Items for Systematic Reviews and Meta-Analyses (PRISMA) and latest Assessment of Multiple Systematic Reviews 2 (AMSTAR2) checklists were used to assess reporting characteristics and methodological quality, respectively.

**Results:**

The literature search yielded 38 potential records, of which five met the inclusion criteria. The total average (SD) for PRISMA was 20.60 (1.14) out of 27. All five SRs have more than one critical weakness in AMSTAR2, so their methodological qualities were considered as critically low. The most frequent problems included nonregistration of study protocol, absence of a list of excluded studies, and unclear acknowledgment of conflicts of interests. The three studies of higher methodological quality reported positive results in pain relief.

**Conclusion:**

The reporting and methodological quality of systematic reviews and meta-analysis studies were suboptimal, which demands further improvement. More efforts are needed to improve validity of systematic reviews and RCTs in this area.

## 1. Background

Dysmenorrhea is one of the most common gynecological conditions in clinic, which has been divided into the primary and secondary types based on different pathogenesis. It is estimated that dysmenorrhea prevalence has varied from 45% to 95%, according to different standards [1]. Primary dysmenorrhea (PD) has affected about 75% reproductive females at certain period of their life. School and work absenteeism caused by severe menstrual pain has been up to 14% [2]. Despite sharing the similar clinical symptoms characterized by cramping pain in the lower abdomen and pelvis before, during, and after period, it is quite different from secondary dysmenorrhea on pathogen and pathogenesis. Secondary dysmenorrhea is usually caused by organic pathological changes including endometriosis, adenomyosis, uterine fibroids, uterine malformation, chronic pelvic inflammation, and interstitial cystitis, while primary one is often in absence of specific organic causes.

Currently, pharmacological therapies for PD include nonsteroidal anti-inflammatory drugs (NSAIDs), which are considered as the first-line treatment for PD according to evidence-based medicine [3]. However, proportional patients turned out to be irresponsive to NSAIDs, which might be due to their complicated pathways and pathological mechanisms [4]. Other treatments include oral hormonal contraceptives [5], intrauterine device, and alternative therapies. Given the fact that 10-20% of females with PD do not respond to NSAIDs or oral contraceptives treatment [1] or are just not suitable for its indications or intolerant to the side effects, alternative therapies have become an important supplement for PD treatment, among which acupuncture has played the leading role. Acupuncture, as an indispensable component of traditional Chinese medicine (TCM), has been widely used in clinical treatment for PD, especially in China. Originated from ancient naive philosophy and empirical medicine, efficacy of acupuncture has been proved in clinical practice. However, no consensus has been reached on its efficacy, and it seems not that acknowledgeable by modern medicine for the moment. Some researchers suggest that acupuncture works through placebo effect instead of its real therapeutic effect [6, 7]. Several SRs, on the top of evidence body pyramid, were carried out to investigate the effect of acupuncture on primary dysmenorrhea, yet gaining inconsistent conclusions [8–10]. Therefore, it is necessary to evaluate the methodological quality and result grade of SRs that included RCT of effect of acupuncture on primary dysmenorrhea. As a result, an overview or umbrella review of SRs is essential to be done to provide stronger evidence for this issue.

## 2. Method

### 2.1. Protocol and Registration

The study protocol has been registered on the PROSPRO platform, and the registration number is CRD42018103334.

### 2.2. Search Strategy for Identification of Studies

We searched three international electronic databases (Cochrane Database of Systematic Reviews, MEDLINE via Ovid, and EMBASE via Ovid) and one Chinese electronic databases (China National Knowledge Infrastructure (CNKI)) to collect potential systematic reviews (SRs) from their inceptions to May 2018. An extensive and comprehensive literature search was conducted without language restriction. Search terms included (systematic review OR meta-analysis) AND (acupuncture OR acupuncture therapy OR acupuncture points OR needle OR electro-acupuncture OR acupuncture analgesia OR warm-acupuncture OR acupressure) AND (dysmenorrhea OR painful menstruation OR menstruation disorder OR period pain OR pelvic pain OR menstruation disturbances), (*系统评价* OR meta*分析*) AND (*针灸* OR *电针* OR *指压*) AND (痛经) with modifications to meet different grammar requirements of each database. The search strategies are listed in Appendix A. In addition, we manually searched through four Chinese journals relevant to acupuncture (Chinese Acupuncture and Moxibustion, the Journal of Clinical Acupuncture and Moxibustion, Acupuncture Research and the Shanghai Journal of Acupuncture and Moxibustion) for related SRs published between 1980 and May 2018 as supplementation. We also checked the reference lists of all relevant SRs identified, and their authors were contacted to identify additional relevant SRs if necessary.

### 2.3. Inclusion and Exclusion Criteria

Population, Intervention, Comparison, Outcome and Study (PICOS) strategy was employed.

#### 2.3.1. Study Participants

Female patients of reproductive age diagnosed with primary dysmenorrhea were eligible. Patients with secondary dysmenorrhea caused by endometriosis, adenomyosis, uterine fibroids, uterine malformation, chronic pelvic inflammation, and other organic pelvic pathological changes were excluded, though it might display similar symptoms as primary one.

#### 2.3.2. Study Intervention

Acupuncture and acupressure were utilized as eligible interventions. Acupuncture requires needle penetrating at acupoints, such as body acupuncture, scalp acupuncture, abdominal acupuncture, ear acupuncture, wrist-ankle needle, or electroacupuncture. Acupressure, as a noninvasive technique, is conducted by pressing the special acupoints with fingers or thumbs. SRs with acupuncture combined therapy or acupuncture-related treatment like point injection, laser acupuncture, transcutaneous electrical nerve stimulation (TENS), cupping, or blood-letting were excluded.

#### 2.3.3. Study Comparison

The control interventions included conventional western medicine treatment like NSAIDs, hormonal birth control, rehabilitation exercise (including physical therapy and occupational therapy), traditional Chinese medicine, acupuncture or acupressure at unrelated acupoints, and sham acupuncture. SRs in which the control group compared different forms of acupuncture treatment would be excluded.

#### 2.3.4. Study Outcome Measures

Primary outcome was menstrual pain intensity measured by visual analog scale (VAS) scores. Secondary outcomes included quality of life and safety. Adverse events such as acupuncture fainting, needle twisting and breaking, bleeding, and organ injury were also taken into account as safety measurement.

#### 2.3.5. Study Design

SRs containing more than one RCT were included. Non-RCT SRs, review comments, overviews of SRs, editorials, and guidelines were excluded.

#### 2.3.6. Eligibility Assessment and Data Extraction

Two reviewers (Z-FR and S-MS) independently screened all the titles and abstracts of retrieved articles and assessed full texts of potential eligibility. Whenever a divergence arose between the two reviewers and could not be settled after discussion, a third reviewer (J-RJ) would recheck the information to make final decision. Older version of duplicate citations was favored for data extraction. A standardized form was designed to extract the following information from each eligible SR: first author, year of publication, country, number of RCTs enrolled, quality assessment tool for RCTs included in SR, and characteristics of interventions in treatment and control groups, including style of acupuncture or acupressure and medicine intake, outcome measures (primary and secondary outcomes), data synthesis methods, main results, and conclusions. This procedure was also conducted separately by two reviewers, and disagreement would be settled by discussion and the introduction of a third reviewer.

#### 2.3.7. SRs Methodological Quality Assessment

“A measurement tool to assess the methodological quality of systematic reviews” (AMSTAR2) was employed to assess methodological qualities of SRs included in this study. AMSTAR is a popular instrument for critically assessing the quality of RCTs included SRs [13]. AMSTAR2 is an update of AMSTAR, which can be used to appraise SRs of both randomized and nonrandomized controlled trials. It has developed its items to 16 from the original 11 and has simpler response categories than the original version, which is more friendly and efficient for users. However, it is not suggested to generate an overall score but to consider potential impact of an inadequate rating for each item. According to AMSTAR2, the 7 critical domains were predefined (Table 1).

Here is the general rule for rating overall confidence in the results of review: SR with no or one noncritical weakness will be rated as high; with more than one noncritical weakness will be rated as moderate; with one critical flaw with or without noncritical weaknesses will be rated as low; with more than one critical flaw with or without noncritical weaknesses will be rated as critically low.

Additionally, Preferred Reporting Items for Systematic Reviews and Meta-Analyses (PRISMA) was applied to assess report quality of SRs.

All eligible articles were assessed by two reviewers by using the two assessment tools above. Discussion and a third reviewer were introduced when confronted with divergences throughout the rating process. The kappa value was used to measure the agreement degree between the two reviewers: kappa value less than 0.4 represented for poor agreement, 0.4 to 0.75 fair agreement, and over 0.75 excellent agreement [14]. Likewise, consensus was acquired by discussion between two reviewers and an independent decision was obtained from a third reviewer (J-RJ) if necessary.

## 3. Results

### 3.1. Results on Literature Search and Selection

**Figure 1:**
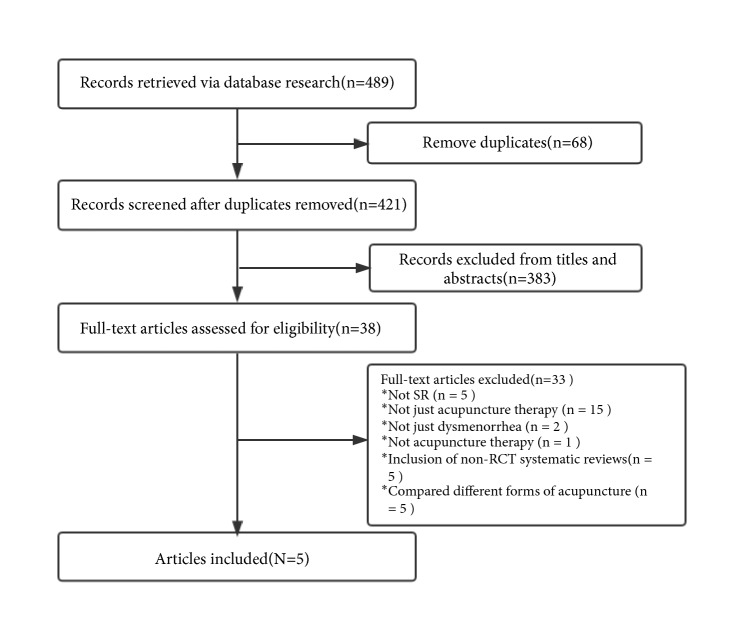
Flow diagram of literature search.

Our searches generated 489 citations, and 68 duplicates were excluded before screening. 383 of the remaining citations were excluded by title and abstract screening. Full texts of the rest 38 citations were retrieved for further assessment, with five eligible articles meeting our inclusion criteria included finally. Here are the reasons for 33 publications' exclusion during the full-text screening process: Five were narrative reviews, 15 were not just acupuncture therapy, two SR were not just dysmenorrhea, one SR was not acupuncture therapy, five included non-RCT systematic reviews, and another five compared different forms of acupuncture. The list of excluded reviews was listed in Appendix B. See details of literature search and SR selection in Figure 1 and Table 2.

### 3.2. Characteristics of Included Reviews

The 5 SRs were all written in English and published between 2010 and 2017. These studies were mainly published by authors from East Asia (four from China and one from South Korea). These reviews reported the results from 248 original RCT studies and 17,392 female patients of primary dysmenorrhea. All the reviews applied Cochrane Handbook for Systematic Reviews of Interventions, Version 5.1.0 for methodological quality assessment of original RCTs in each review. As for interventions, one review focused on electroacupuncture, one observed acupressure, two looked into acupuncture or acupressure at SP6, and the remaining one only focused on acupuncture. Pain relief measured by VAS was considered as primary outcome for five reviews, and secondary outcomes related to life quality varied among different scales, three of which also took adverse effects as secondary outcome. Four reviews employed meta-analysis method as data synthesis, yet the remaining one that did not use it was a narrative systematic review. See Table 2 for full details.

### 3.3. Methodological Appraisal

The assessment results on methodological quality of included reviews were shown in Table 3. According to the evaluation criteria of the latest version of AMSTAR2, since all five SRs had more than one critical weakness (Items 2, 4, 7, 9, 11, 13, and 15), their qualities were considered as critically low.

They all employed the PICO approach (population, intervention, control group, and outcome) as an organizing framework for establishing study questions. Yet no SR provided a protocol registration or publication before commencement of the review (AMSTAR2 Item 2). All reviews had selected study type of only RCT, without explaining specific reasons for selection. Only one review [8] had conducted a comprehensive literature search (AMSTAR2 Item 4). In four reviews [9–12] authors had performed study selection and data extraction in duplicate. Only one review [12] provided a complete list of potentially relevant studies with justification for the exclusion of each (AMSTAR2 Item 7). Five reviews partly provided characteristic information of their included studies. All reviews had evaluated the risk of bias within included RCT studies with Cochrane RoB instruments for RCTs, with two “Yes” and three “Partial Yes” (AMSTAR2 Item 9). None of the reviews had reported founding sources, and only one [8] revealed conflict of interests of the review. Three reviews [9, 11, 12] applied meta-analytical methods appropriately, explaining reasons for fixed or random effects model selection and methods used for heterogeneity investigation. One review [8] which was not applicable for it was a narrative SR without quantitative analysis (AMSTAR2 Item 11). In three reviews [9, 10, 12] authors had evaluated the potential impact of RoB in individual studies on the results of the meta-analysis or other evidence synthesis. Four reviews [8–10, 12] had discussed the impact of RoB in the results interpretation of the review (AMSTAR2 Item 13).Three reviews [8, 11, 12] had explored possibilities of heterogeneity and discussed its impact on the results conclusions and clinical recommendations. Only one [12] had implemented an investigation of publication bias and also discussed its impact on the review results (AMSTAR2 Item 15).

### 3.4. Reporting Quality

**Figure 2:**
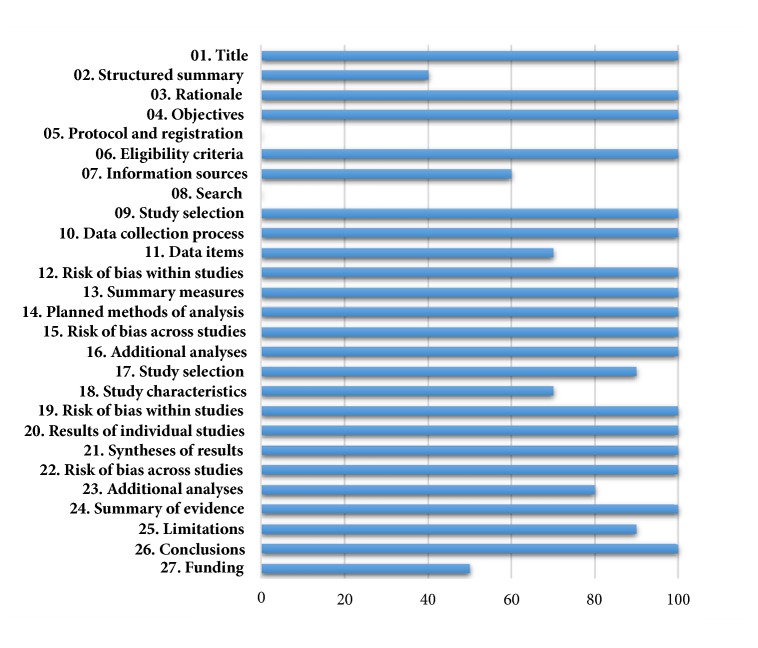
Percentage of reviews that appropriately address each PRISMA element.

The results of the assessment on the reporting of included reviews were shown in Table 4 and Figure 2. Preferred Reporting Items for Systematic Reviews and Meta-Analyses (PRISMA) [15] was applied to assess report qualities of included SRs. The mean (SD) for the PRISMA score was 20.60 (1.14) out of 27. We found that most included reviews are of high reporting quality, and their scores were close to each other. Although the reporting quality was high, there were still some common weaknesses as follows: the trial program did not register in advance and did not provide full search strategy for at least one database; only one study elaborated sources of funding and other support and role of funders for the systematic review. Other items about title, eligibility criteria, study selection, data collection process, risk of bias, summary of evidence, and conclusions were all well reported.

### 3.5. Effectiveness of Acupuncture Therapies

While five systematic reviews reported contradictory results, the three of higher quality [9, 10, 12] suggested that individuals who received acupuncture experienced lower levels of pain than their counterparts who received sham treatments. And two studies [9, 11] on acupressure also reported positive results and improved pain measured with VAS (−1.41cm 95% CI  [−1.61, −1.21]). The highest quality study [12] showed that EA at SP6 was better in pain relief compared to EA at GB39 (*τ*2  =127.47, *χ*2  =79.71, df=5, and *I*2  =94%; MD:11.27; 95% CI: 1.76, 20.78). Cho et al. [8] found that acupuncture was associated with a significant reduction in pain compared with pharmacological treatment or herbal medicine. Besides, one study [11] found acupuncture at different acupoint had no difference in the mean VAS score reduction. Due to the study design, it only demonstrated the effects of difference meridian acupoints on primary dysmenorrhea, which, however, could not rule out the role of acupuncture therapy for the disease.

### 3.6. Adverse Events

Only two studies [8, 10] reported adverse events, such as fainting during acupuncture, a hematoma, and a needling sensation after acupuncture. There were no serious adverse events. But these can be avoided with caution. Therefore, with the correct management and application, acupuncture is a safe technique for PD treatment.

## 4. Discussion

### 4.1. Summary of Main Findings

This overview has provided a summary of effects on pain relief and life quality among females with primary dysmenorrhea treated by acupuncture and acupressure therapies in five eligible SRs of RCTs. This overview included four meta-analyses and one narrative SR, two of which reached positive conclusions [10, 12] while the remaining three proved to be negative [8, 9, 11]. The majority of SRs were considered as relatively high reporting quality and critically low methodological quality, by using the PRISMA and AMSTAR2 tools, respectively.

### 4.2. Suggestions for Better Methodological and Reporting Quality

In this overview, all 5 SRs employed PICO approach in organizing research question and describing inclusion criteria but failed to provide a documented protocol or register information. It is noted that obtaining an open register of a SR in advance is quite essential for conducting a SR. It can help facilitate processing transparency and avoiding post hoc decision bias in methodology [16]. Under the circumstance, registration platform like the practice of the prospective register of systematic reviews in international databases PROSPERO (University of York. Centre for Reviews and Dissemination, Research projects, http://www.crd.york.ac.uk/crdweb, Accessed September 16, 2013) has been emphasized and advocated. The register item has also been recorded in both the PRISMA and AMSTAR2 checklists, indicating its great importance. As a result, review authors should pay more attention to register work and apply for a registration ahead of time.

All included reviews had selected only RCT studies, yet no explaining specific reasons for study type selection. Well-designed and implemented RCTs are considered as gold standards for evaluating interventions for its minimizing or avoiding bias [17]; thus high quality RCT-restricted SRs are able to provide more convincing evidences. Based on AMSTAR2 users guide, review authors should perform study type selection following a general rule that they could ensure whether a systematic review restricted to RCTs would have given a complete summary of the effects of an intervention. For in some cases, there might be enough but unqualified RCTs or inadequate relevant RCTs. In this situation, nonrandomized trials are needed to be added as supplements for generating comprehensive results. Hence, besides stating study types selection, illustrating reasons for selection is of equal importance.

Though all reviews had conducted literature searches in more than one database, however, only one [8] gave comprehensive search strategies. A full and comprehensive literature research is quite critical for secondary literature studies like SRs, which should involve searching in major electronic bibliographic databases like MEDLINE EMBASE and Cochrane Library, registration platforms like PROSPERO, supplemented by checking reference lists of original studies, consulting professional experts, contacting authors or sponsors for full articles or complete data. Grey literatures like dissertations, trials registration, conference abstracts or articles, government policies, and so on are required to cover an all-round search.

Most reviews processed the study selection and data extraction in duplicate, measuring inter-rater agreement by a kappa value and settling discrepancies by discussion and introduction of a third author while the remaining one [8] did not mention this in article. Only one [10] provided a complete list of potential studies with reasons for the exclusion of each. No review had offered adequate details of included studies, mainly lacking information on clinical settings, disease duration, and follow-up visit. Sufficient knowledge on study characters can help review appraisers, readers, and policy-makers to verdict whether the study should be selected or taken as evidence for making clinical practice and health policies. All reviews used Cochrane Collaboration's tool for assessing risk of bias (RoB) of individual RCTs, yet only one review [11] failed to supply ample explanation on the risk of bias when summarizing and interpreting the results.

Apart from the narrative SR [8], the remaining four reviews had all conducted meta-analysis as quantitative synthesis. Only one [11] did not account for RoB in the result. Two reviews [9, 10] failed to explain or discuss heterogeneity in the review results. As for publication bias, only one review [12] reported funnel plot and provided explanation for its possible sources.

No review had reported funding resources and only one [8] stated the conflicts of interests. It is necessary to give details about financial sponsorship and interest disclosure, for some investigations have revealed that commercially sponsored projects may be more inclined to have results and conclusions in favor of their sponsors, which will increase study reporting bias.

PRISMA guideline is mainly responsible for reporting quality assessment. All five reviews acquired a relatively high quality with an average of 20.60/27 scores. Common items with low scores included structured summary, protocol and registration, search strategy, and funding information.

Most weakness can be avoided or declined if review authors could learn and use AMSTAR and PRISMA tools before the commencement, raising their awareness in standard methodology and reporting. The practice of using the widely accepted tools like PRISMA and AMSTAR for designing, reporting, and assessing of systematic reviews needs to be encouraged and advocated, hereby providing more convincing evidence based on their findings.

## 5. Strengths and Limitations

Firstly, it is the latest overview on acupuncture for primary dysmenorrhea, which can provide new evidence reference for clinical practice. Based on the current results and conclusion of high quality SR, the overview suggested that acupuncture may help to alleviate pain caused by primary dysmenorrhea, which could be useful for decision-making for the PD treatment in clinic. Secondly, the employment of new tools for methodological quality assessment AMSTAR2 gives new insight on how to evaluate and improve the methodological quality of SRs.

Despite the strengths, there are also some methodological limitations that might limit the confidence of this overview. For instance, it included only RCT-based systematic reviews, which lacked other types of literatures. Besides, interventions merely contained acupuncture and acupressure, excluding other alternative therapies like moxibustion, tuina, etc. Last but not least, it only took subject VAS as primary outcome measurement, which lacks relatively object items. Though selection of the newest assessment tool AMSTAR2 for quality assessment is a strength of this study, it may also bring some insufficiency. For example, the five included reviews are almost published before the release of AMSTAR2, so some authors failed to follow the rules, which could partially result in low quality for assessment.

## 6. Opportunities for Future Research

By analyzing and pointing out these insufficiencies in these published systematic reviews, we find that the most common problems for included reviews included lack of registration or study protocol, absence of study exclusion list with reasons, and insufficient information on funding sources and interest disclosure and hope this overview can do something for enhancing both reporting and methodological quality of reviews in the future. Applying for a review registration and reporting in a more normative way can greatly improve their reporting quality. Our study has also left some room for undiscussed topics and further development for new overviews, such as taking all complementary therapies as interesting intervention and covering a wider range of literature types, which might bring about new findings.

## 7. Conclusion

There is an increasing number of SRs of acupuncture for PD, and some of them showed potential advantages to acupuncture for PD in pain or related symptoms alleviation. However, many systematic reviews still have methodological flaws which limit their results' confidence. As a result, there are insufficient qualified evidences to determine the effectiveness of acupuncture in the treatment of PD.

## Figures and Tables

**Table 1:** Critical domains based on AMSTAR2.

Critical domain	Context
**Item 2**	Did the report of the review contain an explicit statement that the review methods were established prior to the conduct of the review and did the report justify any significant deviations from the protocol?
**Item 4**	Did the review authors use a comprehensive literature search strategy?
**Item 7**	Did the review authors provide a list of excluded studies and justify the exclusions?
**Item 9**	Did the review authors use a satisfactory technique for assessing the risk of bias (RoB) in individual studies that were included in the review?
**Item 11**	If meta-analysis was performed did the review authors use appropriate methods for statistical combination of results?
**Item 13**	Did the review authors account for RoB in individual studies when interpreting/discussing the results of the review?
**Item 15**	If they performed quantitative synthesis did the review authors carry out an adequate investigation of publication bias (small study bias) and discuss its likely impact on the results of the review?

**Table 2:** Characteristics of systematic reviews.

**First author (year)/country**	**language**	**Number of RCTs included**	**Quality assessment tool for original studies included in SR**	**Intervention**	**Comparisons**	**Primary outcome**	**Adverse effects**	**Data analysis methods**	**Authors conclusions**
Cho 2010 [8]/South Korea	English	27 RCTs	Cochrane Collaboration's tool for assessing risk of bias.	Acupuncture	Placebo control	Pain relief	No serious adverse events	N/A	The evidence for the effectiveness of acupuncture for the treatment of PD is not convincing compared with sham acupuncture.

Chen 2013 [11]/China	English	8 RCTs	Cochrane Collaboration's tool for assessing risk of bias.	Acupuncture /acupressure	Placebo control	Pain relief (VAS)	N/A	Meta- analysis	Acupuncture at the SP6 acupoint may not be more effective in relieving pain than acupuncture at an unrelated acupoint. Acupressure at the SP6 acupoint may provide more effective pain relief than that of control treatment.

Jiang 2013 [9]/China	English	8 RCTs	Cochrane Collaboration's tool for assessing risk of bias.	Acupressure	Placebo control	pain relief (VAS or other scale)	Not reported in review results	Meta- analysis	No convincing evidence for acupuncture in the treatment of PD

Liu 2017 [10]/China	English	23 RCTs	Cochrane Collaboration's tool for assessing risk of bias.	Acupuncture	Placebo control	VAS, a verbal rating scale (VRS), or a numerical rating scale (NRS)	Have 3 side effects, such as fainting during acupuncture, a hematoma, and a needling sensation after acupuncture	Meta- analysis	Acupuncture may be effective for PD

Yu 2017 [12]/China	English	9 RCTs	Cochrane Collaboration's tool for assessing risk of bias.	EA	Placebo control; waiting-list	Pain relief (VAS)	N/A	Meta- analysis	EA can provide considerable immediate analgesia effect for PD.

**Table 3:** Percentage of reviews that appropriately address each AMSTAR2 domain.

**Author **	Q1	**Q2**	Q3	**Q4**	Q5	Q6	**Q7**	Q8	**Q9**	Q10	**Q11**	Q12	**Q13**	Q14	**Q15**	Q16	Ranking of quality
Cho 2010 [8]	Y	N	N	Y	N	N	N	py	py	N	N/A	N/A	Y	Y	N/A	Y	Critically low
Chen 2012 [11]	Y	N	N	N	Y	Y	N	py	py	N	Y	N	N	Y	N	N	Critically low
Jiang 2013 [9]	Y	N	N	N	Y	Y	N	py	Y	N	Y	Y	Y	N	N	N	Critically low
Liu 2017 [10]	Y	N	N	Py	Y	Y	Y	py	py	N	Y	Y	Y	N	N	N	Critically low
Yu 2017 [12]	Y	N	N	py	Y	Y	N	py	Y	N	Y	Y	Y	Y	Y	N	Critically low

**Total**	5	0	0	1	4	4	1	0	1	0	2	3	4	3	0	1	

Abbreviation: Y, yes; PY, partial yes; N, no; N/A, not applicable.

**Table 4:** Reporting quality assessment of systematic reviews by PRISMA.

**No.**	**Section/Topic**		Cho 2010 [8]	Chen 2012 [11]	Jiang 2013 [9]	Liu 2017 [10]	Yu 2017 [12]
1	**TITLE**	Title	Y	Y	Y	Y	Y
2	**ABSTRACT**	Structured summary	Y	N	N	Y	N
3	**INTRODUCTION**	Rationale	Y	Y	Y	Y	Y
4		Objectives	Y	Y	Y	Y	Y
5	**METHODS**	Protocol and registration	N	N	N	N	N
6		Eligibility criteria	Y	Y	Y	Y	Y
7		Information sources	Y	py	py	py	py
8		Search	N	N	N	N	N
9		Study selection	Y	Y	Y	Y	Y
10		Data collection process	Y	Y	Y	Y	Y
11		Data items	py	py	py	Y	Y
12		Risk of bias in individual studies	Y	Y	Y	Y	Y
13		Summary measures	Y	Y	Y	Y	Y
14		Synthesis of results	N/A	Y	Y	Y	Y
15		Risk of bias across studies	Y	Y	Y	Y	Y
16		Additional analyses	Y	Y	Y	Y	Y
17		Study selection	Y	Y	py	Y	Y
18		Study characteristics	Y	py	Y	py	py
19		Risk of bias within studies	Y	Y	Y	Y	Y
20		Results of individual studies	Y	Y	Y	Y	Y
21		Synthesis of results	N/A	Y	Y	Y	Y
22		Risk of bias across studies	Y	Y	Y	Y	Y
23		Additional analysis	N	Y	Y	Y	Y
24	**DISCUSSION**	Summary of evidence	Y	Y	Y	Y	Y
25		Limitations	Y	Y	py	Y	Y
26		Conclusions	Y	Y	Y	Y	Y
27	**FUNDING**	Funding	Y	N	py	py	py

	**TOTAL SCORE**		21	20	19	22	21

**Table 5:** 

**Citation**	**Reason for exclusion**
Armour M, Smith CA. Treating primary dysmenorrhoea with acupuncture: a narrative review of the relationship between acupuncture “dose” and menstrual pain outcomes. Acupuncture in Medicine;34(6):416-24	Not SR
Wang S X, Li Y H, Yin L L. [Advances of studies on treatment of dysmenorrhea with acupoint application][J]. Chinese Acupuncture & Moxibustion, 2005, 25(4):293-295.	Not SR
Yu S, Yang J, Yang M, et al. Application of acupoints and meridians for the treatment of primary dysmenorrhea: a data mining-based literature study[J]. Evidence-Based Complementary and Alternative Medicine,2015,(2015-2-24), 2015, 2015(8):752194.	Not SR
Zhao L, Li P. A survey of acupuncture treatment for primary dysmenorrhea. Journal of Traditional Chinese Medicine 2009;29(1):71-76	Not SR
Song JS, Chen YR, She YF, et al. [Survey on the evaluation indices of acupuncture clinical trials for primary dysmenorrhea in recent 10 years]. Zhongguo Zhenjiu;32(2):187-90	Not SR
Ai-ling, MA Rui-ping, XIAO Wan, et al. Effects of Associated Simple Acupuncture Therapy in the Treatment of Primary Dysmenorrhea: a Systematic Review[J]. J Int Obstet Gynecol, 2014(4):453-458.	Not just acupuncture therapy
CHEN Wen, YU Haihong, LIU Shihong, et al. Systematic Review of Acupuncture Treatment of Primary Dysmenorrhea[J]. CHINESE ARCHIVES OF TRADITIONAL CHINESE MEDICINE, 2013(2):321-325.	Not just acupuncture therapy
Huan Yang, Systematic review of clinical trials of acupuncture related therapies for primary dysmenorrhea[D]. Beijing University of Chinese Medicine, 2008.	Not just acupuncture therapy
Fan Y, Li L, Gong S. Warming acu-moxibustion and moxibustion for primary dysmenorrhea: a systematic review[J]. Chinese Evidence-Based Nursing, 2017.	Not just acupuncture therapy
*刘甜*, 魏华, 徐雪琴,等. *针灸治疗原*发性痛经的 Meta *分析*[J]. 湖南中医*杂志*, 2016, 32(1):143-146.	Not just acupuncture therapy
Kannan P, Claydon LS. Some physiotherapy treatments may relieve menstrual pain in women with primary dysmenorrhea: a systematic review. Journal of physiotherapy;60(1):13-21	Not just acupuncture therapy
Proctor ML, Smith CA, Farquhar CM, et al. Transcutaneous electrical nerve stimulation and acupuncture for primary dysmenorrhea. Cochrane Database of Systematic Reviews (1):CD002123	Not just acupuncture therapy
Xu T, Hui L, Juan Y L, et al. Effects of moxibustion or acupoint therapy for the treatment of primary dysmenorrhea: a meta-analysis.[J]. Alternative Therapies in Health & Medicine, 2014, 20(4):33.	Not just acupuncture therapy
Mojay G. Healing the jade pool—the phyto-aromatic and acupressure treatment of dysmenorrhoea and menopausal syndrome: an East–West approach[J]. International Journal of Aromatherapy, 2002, 12(3):131-141.	Not just acupuncture therapy
Proctor M, Farquhar C, Stones W, et al. Transcutaneous electrical nerve stimulation for primary dysmenorrhoea[M]// The Cochrane Library. John Wiley & Sons, Ltd, 2002:CD002123.	Not just acupuncture therapy
Xu Y, Zhao W, Li T, et al. Effects of acupoint-stimulation for the treatment of primary dysmenorrhoea compared with NSAIDs: A systematic review and meta-analysis of 19 RCTs. BMC complementary and alternative medicine 2017;17(1)	Not just acupuncture therapy
Smith CA, Armour M, Zhu X, et al. Acupuncture for dysmenorrhoea. Cochrane Database of Systematic Reviews 2016;4:CD007854	Not just acupuncture therapy
Chung YC, Chen HH, Yeh ML. Acupoint stimulation intervention for people with primary dysmenorrhea: Systematic review and meta-analysis of randomized trials. Complementary therapies in medicine 2012;20(5):353-63	Not just acupuncture therapy
Smith CA, Zhu X, He L, et al. Acupuncture for primary dysmenorrhoea. Cochrane Database of Systematic Reviews 2011(1):CD007854	Not just acupuncture therapy
Yang H, Liu CZ, Chen X, et al. Systematic review of clinical trials of acupuncture-related therapies for primary dysmenorrhea. Acta obstetricia et gynecologica Scandinavica 2008;87(11):1114-22	Not just acupuncture therapy
Yubin Quan, Chen Lin. A Meta-Analysis of clinical efficacy of acupuncture on dysmenorrhea [J]. clinical Journal of Chinese Medicine, 2016, 8(1):27-28.	Not just primary dysmenorrhea
HUANG Shimin, CHEN Sida, LONG Yongling, et al. Systematic *Ｒ*eview of Abdominal Acupuncture Treatment for Dysmenorrheal, Modern Hospital Dec, 2016, 16(12):1724-1730.	Not just primary dysmenorrhea
Gholami Z. The primary dysmenorrhea and complementary medicine in Iran: A systematic review. International Journal of Fertility and Sterility 2015;9:109 doi: 10.22074/ijfs.2015.4697	Not acupuncture therapy
LIN Han-mei, ZENG Qian-ru, LIU Dan-qing, et al. Meta Analysis of Primary Dysmenorrhea Treated by Acupuncture[J]. HENAN T*Ｒ*ADITIONAL CHINESE MEDICINE, 2015, 35(4):862-865.	Inclusion of non-RCT systematic reviews
Ghiasi A, Keramat A, Mollaahmadi L, et al. The effect of acupressure at the sanyinjiao (Sp6) point on relief of primary dysmenorrhea: A systematic review of clinical trials. Iranian Journal of Obstetrics, Gynecology and Infertility 2017;19(40):55-68	Inclusion of non-RCT systematic reviews
Abaraogu UO, Igwe SE, Tabansi-Ochiogu CS. Effectiveness of SP6 (Sanyinjiao) acupressure for relief of primary dysmenorrhea symptoms: A systematic review with meta- and sensitivity analyses. Complementary therapies in clinical practice;25:92-105	Inclusion of non-RCT systematic reviews
Yang H, Liu CZ, Chen X, et al. Systematic review of clinical trials of acupuncture-related therapies for primary dysmenorrhea. Acta obstetricia et gynecologica Scandinavica 2008;**87**(11):1114-22	Inclusion of non-RCT systematic reviews
Abaraogu U O, Tabansiochuogu C S. As Acupressure Decreases Pain, Acupuncture May Improve Some Aspects of Quality of Life for Women with Primary Dysmenorrhea: A Systematic Review with Meta-Analysis.[J]. Journal of Acupuncture & Meridian Studies, 2015, 8(5):220.	Inclusion of non-RCT systematic reviews
Huang Yijing. Meta-analysis of Treatment Effect of Catgut-embedding in Curing Primary Dysmenorrheal[D]. Guangzhou University of Chinese Medicine, 2015.	Compared different forms of acupuncture
Yafeng Wang. Acupuncture at Sanyinjiao (SP6) for the Treatment of Primary Dysmenorrhea: A Meta-Analysis [A]. Beijing University of Chinese Medicine (BUCM), China Association for Acupuncture and Moxibustion (CAAM), 2016:2.	Compared different forms of acupuncture
LI Ge, SI Jinhua, ZHAO Chen, et al. Network meta-analysis on clinical effects of acupuncture in treatment of primary dysmenorrhea[J]. CHINESE JOURNAL OF EVIDENCE-BASED MEDICINE, 2017(10):1212-1223.	Compared different forms of acupuncture
Sun J, Wang Y, Zhang Z, et al. [Efficacy of filiform needle manipulation on primary dysmenorrhea:a systematic review]. Zhongguo Zhenjiu 2017;37(8):887-92	Compared different forms of acupuncture
Wang Y, Sun J, Zhang Z, et al. [Impact of deqi on acupoint effects in patients with primary dysmenorrhea:a systematic review of randomized controlled trials]. Zhongguo Zhenjiu 2017;37(7):791-97	Compared different forms of acupuncture

Full-text articles excluded(n=33)

*∗*Not SR (n = 5)

*∗*Not just acupuncture therapy (n = 15)

*∗*Not just primary dysmenorrhea (n = 2)

*∗*Not acupuncture therapy (n = 1)

*∗*Inclusion of non-RCT systematic reviews(n = 5)

*∗*Compared different forms of acupuncture (n = 5)

## Appendix

### A.

#### A.1. EMBASE

  Search Strategy:
  #1. review:it(1690165)
  #2. “systematic review”(112260)
  #3. “meta analysis”(122870)
  #4. “systematic*∗* review*∗*”(128105)
  #5. “meta analy*∗*”(142180)
  #6. #1 OR #2 OR #3 OR #4 OR #5(1833384)
  #7. acupuncture(33409)
  #8. “acupuncture therapy”/exp(29773)
  #9. “acupuncture points”/exp(1688)
  #10. “electroacupuncture”/exp(4344)
  #11. “acupressure*∗*”/exp(1533)
  #12 “Acupuncture Analgesia”/exp(1292)
  #13. #7 OR #8 OR #9 OR #10 OR #11 OR #12 OR #13(33491)
  #14. “menstruation Disorder*∗*”/exp(5472)
  #15. “dysmenorrhea”/exp(10241)
  #16. “menstruation disturbances”/exp(52518)
  #17. “pelvis pain”/exp(17996)
  #18. “painful menstruation”/exp(104)
  #19. “painful period”/exp(33)
  #20. “period pain”/exp(333)
  #21. Dysmenorrh*∗*/exp(10558)
  #22. #14 OR #15 OR #16 OR #17 OR #18 OR #19 OR #20 OR #21(68191)
  #23. #6 AND #13 AND #22(328)

#### A.2. MEDLINE

  Database: Ovid MEDLINE(R) In-Process & Other Non-Indexed Citations, Ovid MEDLINE(R) Daily and Ovid MEDLINE(R) <1946 to Present> Search Strategy:
  #1. review:it(2390294)
  #2. “systematic review”(110122)
  #3. “meta analysis”(110031)
  #4. “systematic*∗* review*∗*”(125848)
  #5. “meta analy*∗*”(128332)
  #6. #1 OR #2 OR #3 OR #4 OR #5(2477564)
  #7. acupuncture(27634)
  #8. “acupuncture therapy”/exp(15022)
  #9. “acupuncture points”/exp(6135)
  #10. “electroacupuncture”/exp(4639)
  #11. “acupressure*∗*”/exp(1125)
  #12 “Acupuncture Analgesia”/exp(1495)
  #13. #7 OR #8 OR #9 OR #10 OR #11 OR #12 OR #13(28940)
  #14. “menstruation Disorder*∗*”/exp(2064)
  #15. “dysmenorrhea”/exp(5751)
  #16. “menstruation disturbances”/exp(7049)
  #17. “pelvis pain”/exp(9787)
  #18. “painful menstruation”/exp(92)
  #19. “painful period”/exp(30)
  #20. “period pain”/exp(254)
  #21. Dysmenorrh*∗*/exp(6390)
  #22. #14 OR #15 OR #16 OR #17 OR #18 OR #19 OR #20 OR #21(22518)
  #23. #6 AND #13 AND #22(111)

### B.

See Table 5.
